# Complete Chloroplast Genome Sequences and Comparative Analysis of *Chenopodium quinoa* and *C. album*

**DOI:** 10.3389/fpls.2017.01696

**Published:** 2017-10-06

**Authors:** Su-Young Hong, Kyeong-Sik Cheon, Ki-Oug Yoo, Hyun-Oh Lee, Kwang-Soo Cho, Jong-Taek Suh, Su-Jeong Kim, Jeong-Hwan Nam, Hwang-Bae Sohn, Yul-Ho Kim

**Affiliations:** ^1^Highland Agriculture Research Institute (HARI), National Institute of Crop Science, Rural Development Administration, Pyeongchang, South Korea; ^2^Department of Biological Sciences, Kangwon National University, Chuncheon, South Korea; ^3^Phygen Genomics Institute, Seongnam, South Korea

**Keywords:** Chenopodioideae, chloroplast genome, phylogenetic tree, InDel, tandem repeats

## Abstract

The *Chenopodium* genus comprises ~150 species, including *Chenopodium quinoa* and *Chenopodium album*, two important crops with high nutritional value. To elucidate the phylogenetic relationship between the two species, the complete chloroplast (cp) genomes of these species were obtained by next generation sequencing. We performed comparative analysis of the sequences and, using InDel markers, inferred phylogeny and genetic diversity of the *Chenopodium* genus. The cp genome is 152,099 bp (*C. quinoa*) and 152,167 bp (*C. album*) long. In total, 119 genes (78 protein-coding, 37 tRNA, and 4 rRNA) were identified. We found 14 (*C. quinoa*) and 15 (*C. album*) tandem repeats (TRs); 14 TRs were present in both species and *C. album* and *C. quinoa* each had one species-specific TR. The *trnI-GAU* intron sequences contained one (*C. quinoa*) or two (*C. album*) copies of TRs (66 bp); the InDel marker was designed based on the copy number variation in TRs. Using the InDel markers, we detected this variation in the TR copy number in four species, *Chenopodium hybridum, Chenopodium pumilio, Chenopodium ficifolium*, and *Chenopodium koraiense*, but not in *Chenopodium glaucum*. A comparison of coding and non-coding regions between *C. quinoa* and *C. album* revealed divergent sites. Nucleotide diversity >0.025 was found in 17 regions—14 were located in the large single copy region (LSC), one in the inverted repeats, and two in the small single copy region (SSC). A phylogenetic analysis based on 59 protein-coding genes from 25 taxa resolved Chenopodioideae monophyletic and sister to Betoideae. The complete plastid genome sequences and molecular markers based on divergence hotspot regions in the two *Chenopodium* taxa will help to resolve the phylogenetic relationships of *Chenopodium*.

## Introduction

Chloroplast (cp) is a plant organelle involved in photosynthesis that has originated from an ancestral endosymbiotic cyanobacteria (Cho et al., 2015). This organelle plays a role in photosynthetic carbon fixation, providing essential energy to plants (Raven and Allen, 2003). In angiosperms, the chloroplast genome consists of a circular DNA molecule with quadripartite structure comprised of a pair of inverted repeats (IRs), one large single copy region (LSC), and one small single copy region (SSC; Chaney et al., 2016; Cho et al., 2016; Fu et al., 2016). In addition to a quadripartite structure, the chloroplast genome contains about 100–130 genes with highly conserved order and sequences among most land plants (Smith, 2015). Due to its highly conserved sequence, compact size, lack of recombination, and maternal inheritance, the cp genome has been used for generating genetic markers for phylogenetic classification (Choi et al., 2016; Hu et al., 2016), divergence dating (Krak et al., 2016), and DNA barcoding system for molecular identification (Dong et al., 2012). Especially, low evolutionary rate of the cp genome in taxa that are not very young makes it an ideal system for assessing plant phylogeny (Smith, 2015). Sequencing of the complete cp DNA genome began in 1991 (Taberlet et al., 1991) and until present days, the cp genomes from 1,200 species of algae and plants have been sequenced (http://www.ncbi.nlm.nih.gov/genome/organelle/).

*Chenopodium* sensu lato belongs to the subfamily Chenopodioideae (Amaranthaceae, Caryophyllales), and it is the second largest and taxonomically complex genus (Rahiminejad and Gornall, 2004). The traditional family Chenopodiaceae comprised about 100 genera and 1,700 species, mainly distributed in temperate and subtropical regions. However, at present, based on molecular evidence, the family is recognized as the subfamily Chenopodioideae within Amaranthaceae and many of its genera are classified within separate subfamilies of the amaranth family (The Angiosperm Phylogeny Group, 2016). Although *Chenopodium* is considered monophyletic within Chenopodioideae, some researchers reported the genus polyphyletic (Fuentes-Bazan et al., 2012a,b). In addition, taxonomic identification of *Chenopodium* has been controversial because of the highly polymorphic leaf shape, floral structure, and seed morphology (La Duke and Crawford, 1979; Kurashige and Agrawal, 2005).

*Chenopodium* species are cultivated worldwide not only as pseudocereals but also as leafy vegetables. Among them, *Chenopodium quinoa* and *Chenopodium album* are most important species grown as grain and vegetable crops, respectively. *C. album* is an important source of vitamins and micronutrients in India (Bhargava et al., 2007), but also one of the worst weeds. Quinoa is an annual plant that originated from the Andean region and whose worldwide cultivation has been increasing rapidly (Jacobsen et al., 2003). Quinoa is recognized as a crop of great value for its high abiotic stress tolerance and high nutritious content (Repo-Carrasco et al., 2003; Choukr-Allah et al., 2016; Filho et al., 2017).

Several recent studies have attempted to elucidate the origin and polyploidization of the genome in *C. album*, an allohexaploid formed by hybridization between diploid and tetraploid taxa (Krak et al., 2016). The complete nuclear genome sequence of the tetraploid *C. quinoa* (2*n* = 4*x* = 36) was reported at 1.39 gigabases with chromosome specific scale reference genome sequences (Jarvis et al., 2017). In contrast, the chloroplast genome sequence in *Chenopodium* has remained incomplete until now since only a few reports provide information about chloroplast genes such as the non-coding *rpl32-trnL* region (Krak et al., 2016) and the *rbcL* (Kadereit et al., 2003) and *matK/trnK* genes (Fuentes-Bazan et al., 2012b).

In the present study, we report a high quality complete chloroplast genome sequences of the two important agronomic *Chenopodium* species, *C. album* and *C. quinoa*, obtained with the next generation sequencing technology. In addition, we conducted a comparative genomic analysis using tandem repeats, InDels, simple sequence repeats (SSRs) polymorphism, and genetic diversity to identify valuable markers for DNA barcoding and phylogenetic analysis. Additionally, we developed and applied InDel markers based on the variation in tandem repeats (TRs) copy number in *trnI-GAU* intron sequence as a possible DNA marker in other species of Chenopodioideae for phylogenetic analysis.

## Materials and methods

### Plant material

Genetic resources of *Chenopodium quinoa* (8 accessions) were obtained from the National Agrobiodiversity Center of the Rural Development Administration (http://genebank.rda.go.kr), Korea, and cultivated and harvested in the Highland Agriculture Research Institute (800 m above sea level), Pyeongchang, Korea (Table S1). Leaves of *C. album* and five other *Chenopodium* species were collected from the specimens deposited at the Kangwon National University Herbarium (KWNU; Table S1).

### Chloroplast genome sequence assembly

Total genomic DNA was extracted from ~100 mg of fresh or dry leaves removed from a single plant using a NucleoSpin Plant II kit (Macherey-Nagel, GmbH, Düren, Germany) following manufacturer's instructions. Paired-end libraries of *C. quinoa* and *C. album* were constructed with an Illumina Paired-End DNA library Kit (San Diego, CA, USA) according to manufacturer's protocol and sequenced using the Illumina genome analyzer (Hiseq200) platform at Macrogen (http://www.macrogen.com/ko/). The chloroplast (cp) genome assembly was conducted by the *de novo* assembly protocol (Cho et al., 2015) via the Phyzen bioinformatics pipeline (http://phyzen.com). Briefly, a 500-bp paired-end library (approximate insert size 350–450 bp) generated 9,086,336 reads from *C. quinoa* and 6,991,000 reads form *C. album*. Low quality sequences (Phred score < 20) were trimmed using CLC Genomics Workbench (version 6.04; CLC Inc., Arhus Denmark). After trimming, the libraries for *C. quinoa* and *C. album* included 8,121,007 and 6,433,359 reads, respectively. Then, the *de novo* assembly was implemented using the CLC Genome Assembler (http://www.clcbio.com/products/clc-assembly-cell). A total of 1,190,359 and 383,862 reads were aligned and selected using nucmer tool in MUMmer (Delcher et al., 2003) and *Spinacia oleracea* sequence (NC_002202) as a reference. The draft cp genome contigs were merged into a single contig by joining overlapping terminal sequences of each contig. The extracted cp genomes of *C. quinoa* and *C. album* were 152,099 and 152,167 bp, with a mean coverage of 1,840 X and 645 X, respectively. The complete cp genome sequence was annotated using DOGMA (Wyman et al., 2004) and manual editing through comparison with the reported cp genomes of the reference species *S. oleracea* (NC_002202). Circular maps of the cp genome were generated using OGDraw v1.2 (Lohse et al., 2013).

### Comparative analysis and divergence hotspot identification

mVISTA was used to compare similarities between two *Chenopodium* species (Mayor et al., 2000). Nucleotide and amino acids diversity was analyzed by BLASTN and BLASTP, and TRs were analyzed using Tandem Repeat Finder (Benson, 1999) with advanced parameters. The alignment parameters, match, mismatch, indels, were set to 2, 7, 7, respectively; the minimum alignment score to report repeats was 50; the minimum length was 6 bp; and the motif identity percent was 100%. The simple sequence repeats were detected using IMEx (www.mcr.org.in/IMEX; Mudunuri and Nagarajaram, 2007) with minimal repeat numbers of 10, 5, 4, 3, 3, and 3 for mono-, di-, tri-, tetra-, penta-, and hexa-nucleotides, respectively. The substitution rates *Ks* and *Ka* were calculated with PAL2NAL (Suyama et al., 2006). Chloroplast genome sequences of two *Chenopodium* species (*C. quinoa* and *C. album*) were aligned using MAFFT (Katoh et al., 2002), and nucleotide diversity (*Pi*) and the total number of mutations (*Eta*) were determined using DnaSP (Librado and Rozas, 2009).

### Phylogenetic analysis

For phylogenetic analyses, two datasets were created. One dataset comprised sequences of 59 protein-coding genes from 25 Caryophyllales plants; the ingroup included 1 Aizoaceae, 1 Cactaceae, 11 Caryophyllaceae, and 11 Amaranthaceae, and *Fagopyrum tataricum* (Polygonaceae) was used as the outgroup (Table S2). The second dataset comprised the *trnI-GAU* intron sequences of seven *Chenopodium* species and one outgroup (*S. oleracea*). The sequences in both data matrices were compiled and aligned with MAFFT (Katoh et al., 2002). The maximum likelihood analyses of both data matrices were performed using RAxML v7.4.2 with 1,000 bootstrap replicates and the GTR+I+G model (Stamatakis, 2006). This substitution model was chosen under Akaike information criterion (AIC) and Akaike information criterion with correction (AICc) in jModeltest v. 2.1.10 (Darriba et al., 2012).

### PCR amplification using InDel markers

The total genomic DNA was used for PCR amplification with InDel specific primers (Table S6). The PCR reactions (20 μL) included 10 ng of genomic DNA and the AccuPower PCR PreMix (Bioneer, Daejeon, Korea) consisting of 0.2 U/μL *TOP* DNA polymerase, 1.5 mM Mg^2+^, and 250 μM of dNTP mixture with 5 pMol of each primer. The PCR amplification was performed in a thermocycler (ProFlex PCR System, Applied Biosystems, Foster City, CA, USA) using the following cycling parameters: initial denaturation at 94°C for 4 min, followed by 25 cycles of 94°C for 30 s, 65°C for 30 s, and 72°C for 1 min, and a final extension at 72°C for 7 min. The PCR products were analyzed by electrophoresis on 1.8% agarose gels and sequenced by direct sequencing at Bioneer Co. (Daejeon, Korea).

## Results

### Complete chloroplast genome sequences

**Figure 1:**
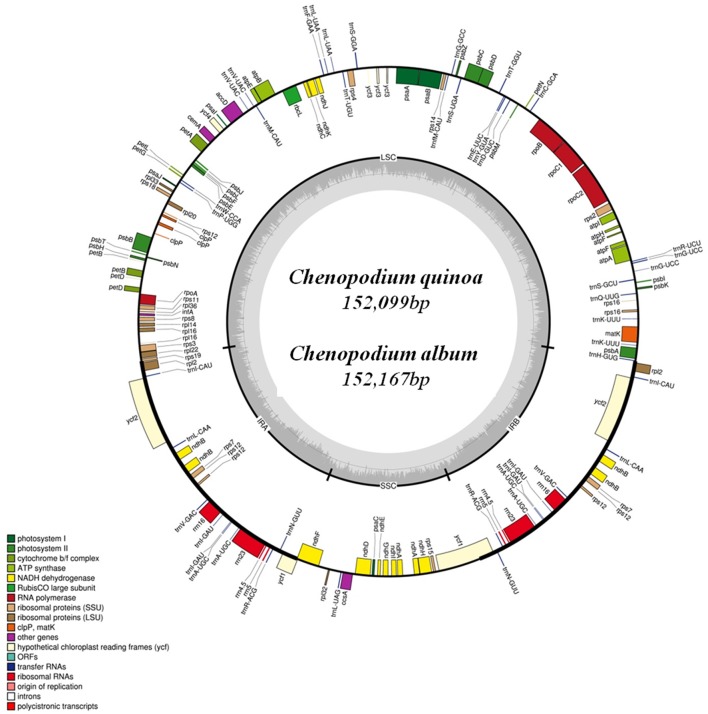
The chloroplast genome map of *Chenopodium quinoa* and *C. album*. Genes shown inside the circle are transcribed clockwise, and those outside the circle are transcribed counterclockwise.

The complete cp genome of *C. quinoa* and *C. album* consisted of a single circular molecule with quadripartite structure (Figure 1). The size of the *C. quinoa* and *C. album* cp genomes was 152,099 bp and 152,167 bp, respectively. They consisted of a pair of IRs (IRa and IRb) 25,205 and 25,193 bp long, respectively, separated by the LSC (83,582 and 83,676 bp), and one SSC (18,107 and 18,105 bp) region (Table 1). The genomes contained 78 coding genes, accounting for 79,115 and 78,930 bp of the *C. quinoa* and *C. album* cp genome, respectively; of those, 62, 5, and 11 genes were located in the LSC, IR, and SSC region, respectively (Table S3). The total length of coding sequences (CDS) was 79,115 bp (the average CDS length was 849 bp) in *C. quinoa* and 78,930 bp (the average CDS length of 847 bp) in *C. album*. The total number of RNA bases was 11,906 (in *C. quinoa*) and 11,835 (in *C. album*), and the overall GC-content was similar in both species, about 37.2%. A sequence inversion was detected in the *rbcL-trnV* region (about 3.1 kb) compared to the *S. oleracea* cp genome (Figure S1). The complete cp genomes of *C. quinoa* and *C. album* are deposited in the GenBank under the accession numbers KY419706 and KY419707, respectively (Table S2).

**Table 1:** Comparison of the complete chloroplast genome between *Chenopodium quinoa* and *C. album*.

**Features**	***C. quinoa* (GQ9)**	***C. album* (KWNU-15)**
Total Sequence Length (bp)	152,099	152,167
Large Single Copy (bp)	83,582	83,676
Inverted Repeat Region (bp)	50,410	50,386
Small Single Copy (bp)	18,107	18,105
GC Content (%)	37.24	37.25
Protein-Coding Genes	78	78
Total CDS^a^ Bases (bp)	79,115	78,930
Average CDS Length (bp)	849.45	847.54
Total RNA Bases (bp)	11,906	11,835
Total Tandem Repeat Length (bp)	938	1,066
Total Simple Sequence Repeat (bp)	486	586
Average Tandem Repeat Length (bp)	67.00	71.06
Average Intergenic Distance (bp)	206.08	207.18

a *CDS, coding sequences*.

### Gene contents and hotspot region in cp genomes

**Figure 2:**
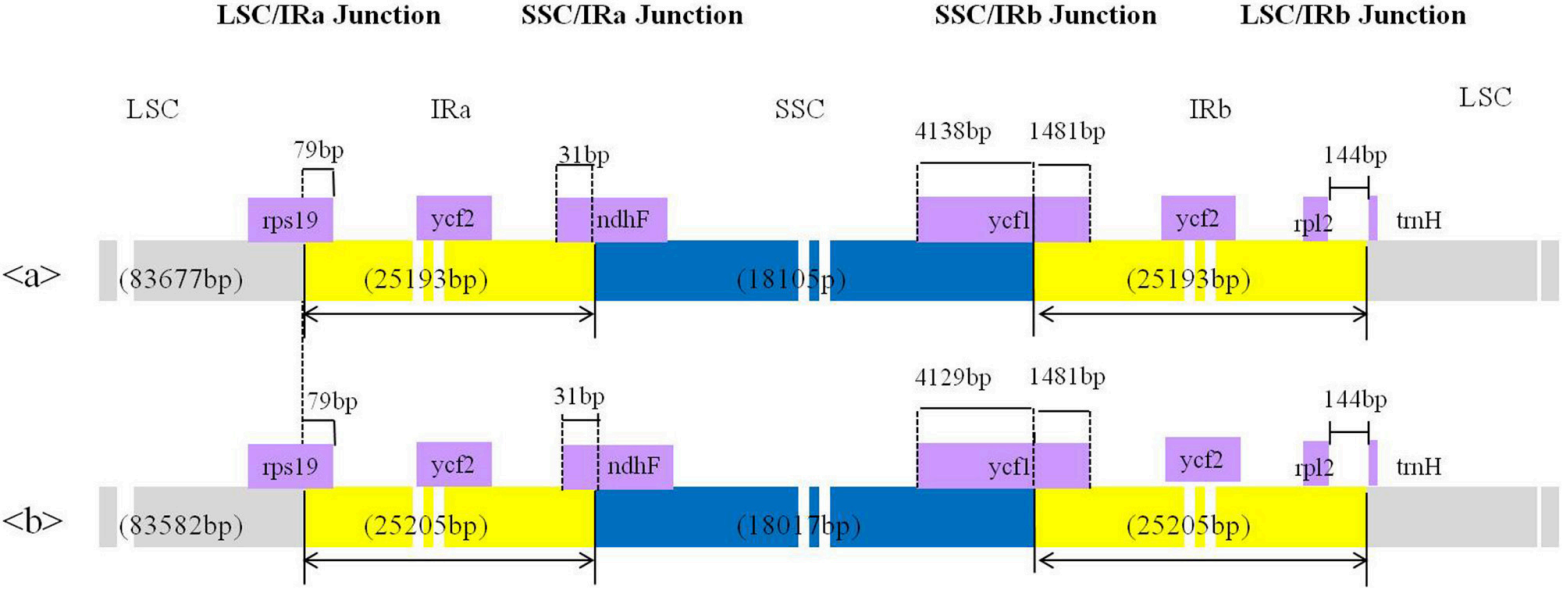
Comparison of the borders of the large single copy (LSC), small single copy (SSC), and inverted repeat (IR) regions of the chloroplast genome between two *Chenopodium* species. a, *Chenopodium album;* b, *C. quinoa*.

**Figure 3:**
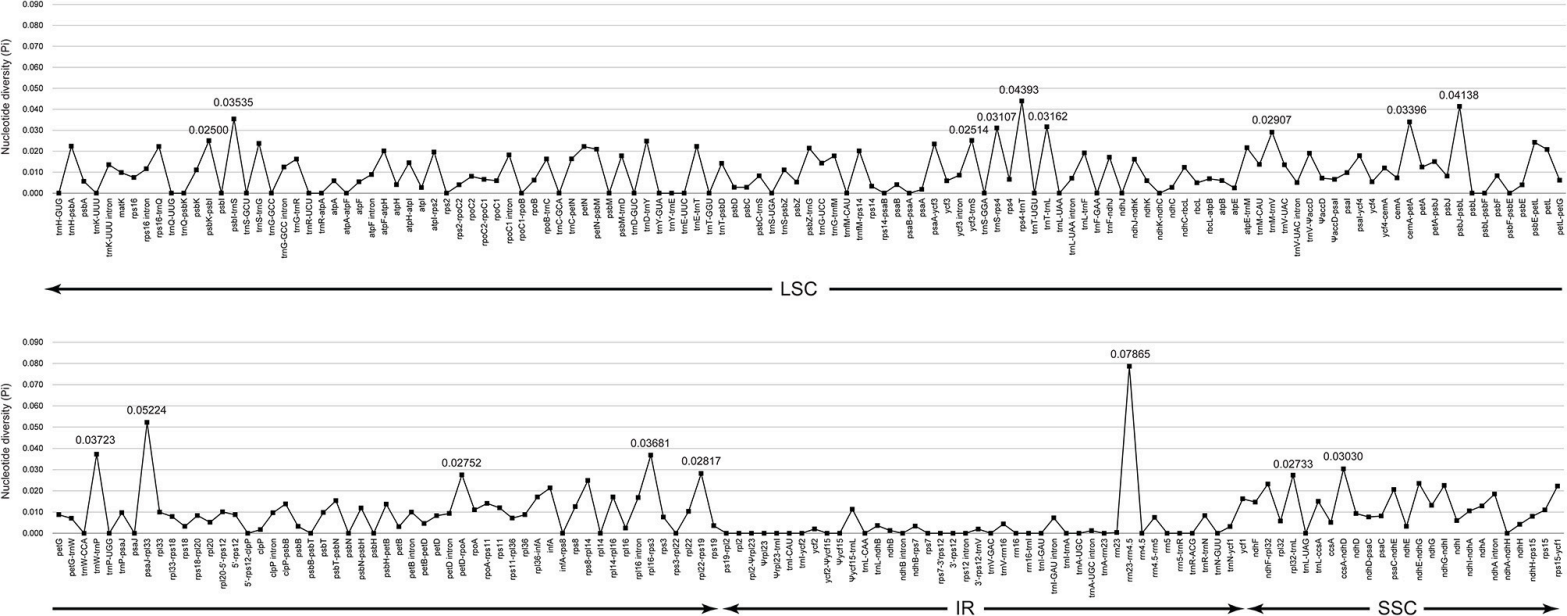
Comparison of the nucleotide diversity (*Pi*) values between *Chenopodium quinoa* and *C. album*.

The complete cp genomes of *C. quinoa* and *C. album* were compared and analyzed. The gene content, order, and orientation in the cp genomes of the two species were similar (Figure 1). The coding regions in both species were highly conserved, except for *matK* gene with 98.2% homology at the amino acid level (Figure S2; Table S3). The overall identity of nucleotides and amino acid sequences of coding genes was 99.8 and 99.7%, respectively, with the IR region having the lowest identity (Table S3). In general, the IR region is known to be more conservative than the LSC and SSC regions. However, this is a trend when comparing the entire IR region to the entire LSC or SSC regions. In addition, nucleotide diversity of some genes or IGS in the IR region can be higher than that of the LSC or SSC regions (Yang et al., 2016; Park et al., 2017; Song et al., 2017). Due to highly conserved coding regions, the *Ka*/*Ks* ratio was very low, approaching zero. However, the *Ka*/*Ks* values for some genes, including *matK, rps16, rpoC2, ycf1*, and *ycf 2*, were higher (Table S3). The IR/LSC and IR/SSC junction regions were compared to identify the IR expansion or contraction. The *rps19, ndhF, ycf1, rpl2*, and *trnH* genes were located in the junctions of the LSC/IRa, IRa/SSC, SSC/IRb, and IRb/LSC regions, respectively; the border position in *C. quinoa* was the same as that in *C. album*, which implied no IR expansion or contraction (Figure 2). The coding regions, introns, and intergenic spacer were compared between the two *Chenopodium* species. The sequence divergence between *C. quinoa* and *C. album* ranged from 0 to 0.07865. The IR region was much more conserved compared to the LSC and SSC regions. Seventeen regions, *psbK-psbI, psbI-trnS, ycf3-trnS, trnS-rps4, rps4-trnT, trnT-trnL, trnM-trnV, cemA-petA, psbJ-psbL, trnW-trnP, psaJ-rpl33, petD-rpoA, rpl16-rps3, rpl22-rps19, rrn23-rrn4.5, ccsA-ndhD*, and *rpl32-trnL*, showed high levels of sequence variation (exceeding 0.025). Of those, 14 regions were located in the LSC, one in the IR, and two in the SSC (Figure 3; Table S4).

### Tandem repeats, InDels, and SSR characteristics

The number, length, and repeat unit of TRs were similar and highly conserved in both species, except for the copy number variation. A total of 14 and 15 TRs, 938 bp and 1,066 bp in length, were identified in the cp genomes of *C. quinoa* and *C. album*, respectively (Table 1). The average TR length was 71 bp in *C. album*, 4 bp longer than that of TRs in *C. quinoa*. Among TRs, nine TRs were located in the IR, four within the LSC, and three in the SSC region (Table 2) in *C. album*. One specific TR (24 bp) detected in intergenic sequences between *rps12* and *petB* of the LSC region in *C. album* was absent in *C. quinoa*; the two species shared 14 TRs in their cp genomes; one TR (64 bp) was only found in *C. quinoa* between *rrn4.5* and *rrn5* intergenic sequences (Table S5). We identified one more copy number in three TRs (TR2, TR8, and TR10) in the *C. album* cp genome compared to that of *C. quinoa* (Table 2).

**Table 2:** Variations in tandem repeat number of chloroplast genome sequences between *Chenopodium quinoa* and *C. album*.

**Tandem repeat**	**Position^z^**	**Repeat unit length (bp)**	**Repeat units sequences**	**Repeat numbers of *C. quinoa/C. album***	**Region^y^**	**Remark**
TR1	IGS (*atpH-atpI*)	13	ATAGAATATCTTG	4/4	LSC	
TR2	IGS (*trnE-trnT*)	18	ATTAATAATTAATCGAAT	3/4	LSC	
TR3	IGS (*rps12-petB*)	12	TTTTTATCCCCT	0/2	LSC	
TR4	IGS (*petB-petD*)	17	AATTTTATATTTAGTTA	2/2	LSC	
TR5	IGS (*rpl2-trnI*)	24	AGTTCGAGTTTCAATAAGAATGCT	2/2	IR	
TR6	IGS (*rpl2-trnI*)	51	ATGAGTTCGAGTTTCAATAAGAATGCTAGTTCTTACTGTTCATATGTTATG	2/2	IR	
TR7	G (*ycf2*)	21	TTTGTCCAAGTCACTTCTCTT	4/4	IR	
TR8	G (*ycf2*)	18	TATTGATGCTAGTGACGA	4/5	IR	
TR9	IGS (*rps12-trnV*)	18	TTTTCTATTAGATTAGTA	2/2	IR	
TR10	G (*trnI-GAU*)	66	GCAATTTTGCAAAAGGATCTTCAAATTCTTTCTGGAGGACTGCAAATCCTTTCTTAGGAAGAACTT	1/2	IR	Indel_QA_02
TR11	G (*trnI-GAU*)	95	AAATTCTTTCTGGAGGACTGAAAATCCTTTCTTAGGAAGAACTTGCAATTTTTTCTCTAGACTCGAAATGGGAGCAAGTTTGAAAAAGGATCTTC	2/2	IR	
TR12	IGS (*rrn4.5-rrn5*)	32	CATTGGTCAACTCTTTGACAACACGAAAAAAC	2/2	IR	
TR13	IGS (rrn5-rrn23)	32	TGGTTTTTTCATGTTGTCAAAGAATTGAACAA	2/0	IR	
TR14	G (*ndhF*)	21	AATAAAAACCTAAAATCTCCT	2/2	SSC	
TR15	IGS (*ndhF-rpl32*)	24	TAATGAAAAAAATAAATTTATTAT	2/2	SSC	
TR16	G (*ycf1*)	21	TTTTGATTATTG	2/2	SSC	

z *IGS, Intergenic sequence; G, Genic sequence*.

y *LSC, Large Single Copy; IR, Inverted repeat; SSC, Small single copy*.

**Figure 4:**
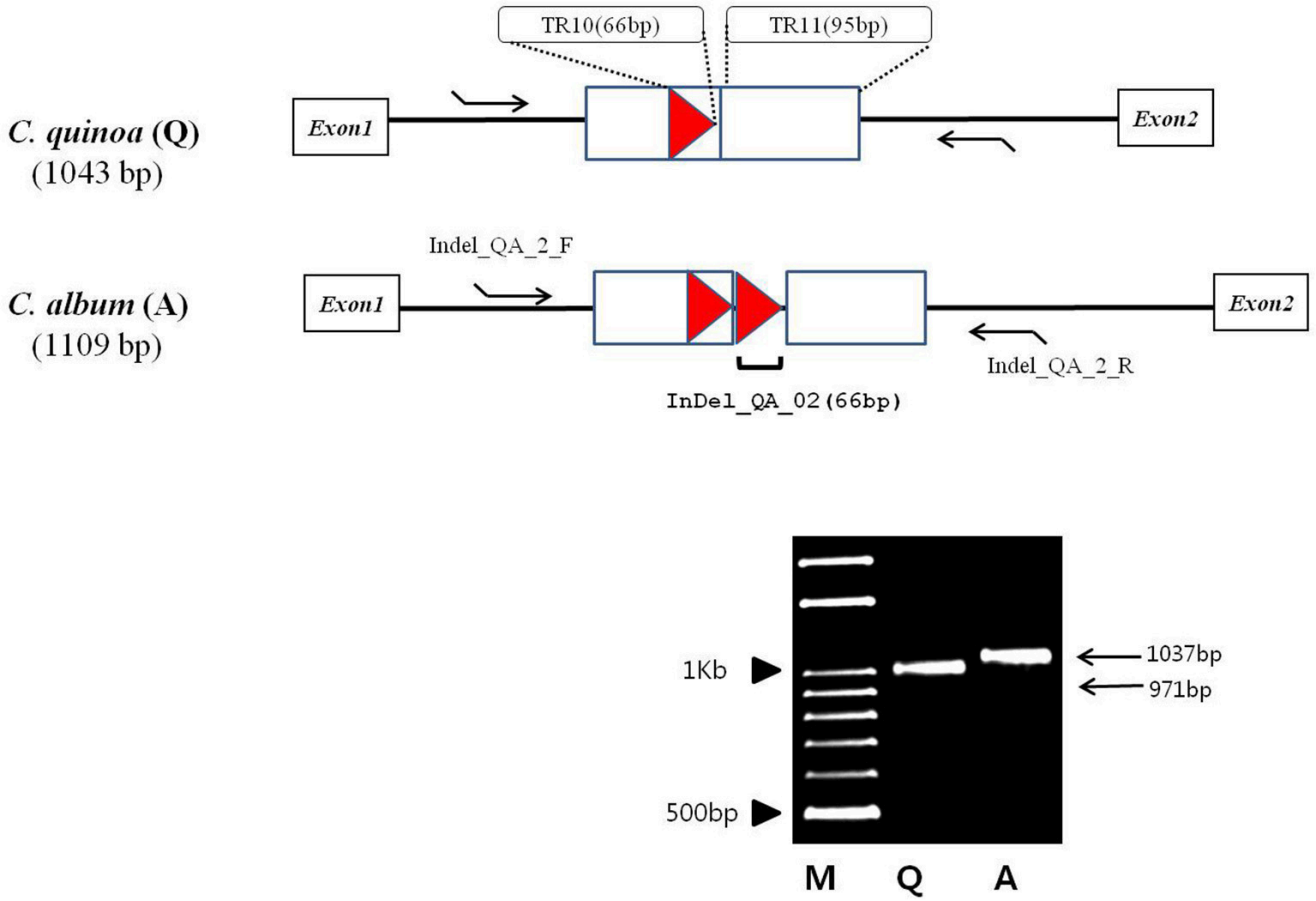
Schematic diagram of the alignment of the *Chenopodium quinoa* (Q) and *C. album* (A) *trnI-GAU* gene sequences. Tandem repeats, 95 and 66 bp long, are designated with a rectangle and a triangle, respectively. Tandem repeat motives and copy numbers are shown in Table S5. InDel_QA_02 primers (Table S6) that amplify the 66 bp tandem repeat region are shown as arrows. M, 100 bp DNA ladder; Q, *C. quinoa*; A, *C. album*.

Most of the InDels were found in the IR region; two InDels (both longer than 60 bp) in the two species were located in the coding sequences of *ycf2* and *trnI-GAU* and were 90 and 66 bp long, respectively (Table S6). We detected quite an interesting variation in the copy number of the *trnI-GAU* intron sequence between exon 1 and exon 2. Namely, *C. quinoa* and *C. album* had the same copies of TR11, both 95 bp long, whereas *C. album* had two copies of TR10 within the *trnI-GAU* intron compared to only one copy in *C. quinoa*, which accounted for the 66 bp long InDel designated InDel_QA_02 (Figure 4). We designed InDel specific primers to confirm the InDel in the *trnI-GAU* intron sequence by PCR amplification in both species (Table S6). The size variation of the resulting amplicons showed an exact 66 bp difference between the two species (Figure 4) and dot-plot analysis of the aligned sequences of InDel_QA_02 confirmed a 66 bp InDel in *trnI-GAU* intron sequences (Figure S3).

**Figure 5:**
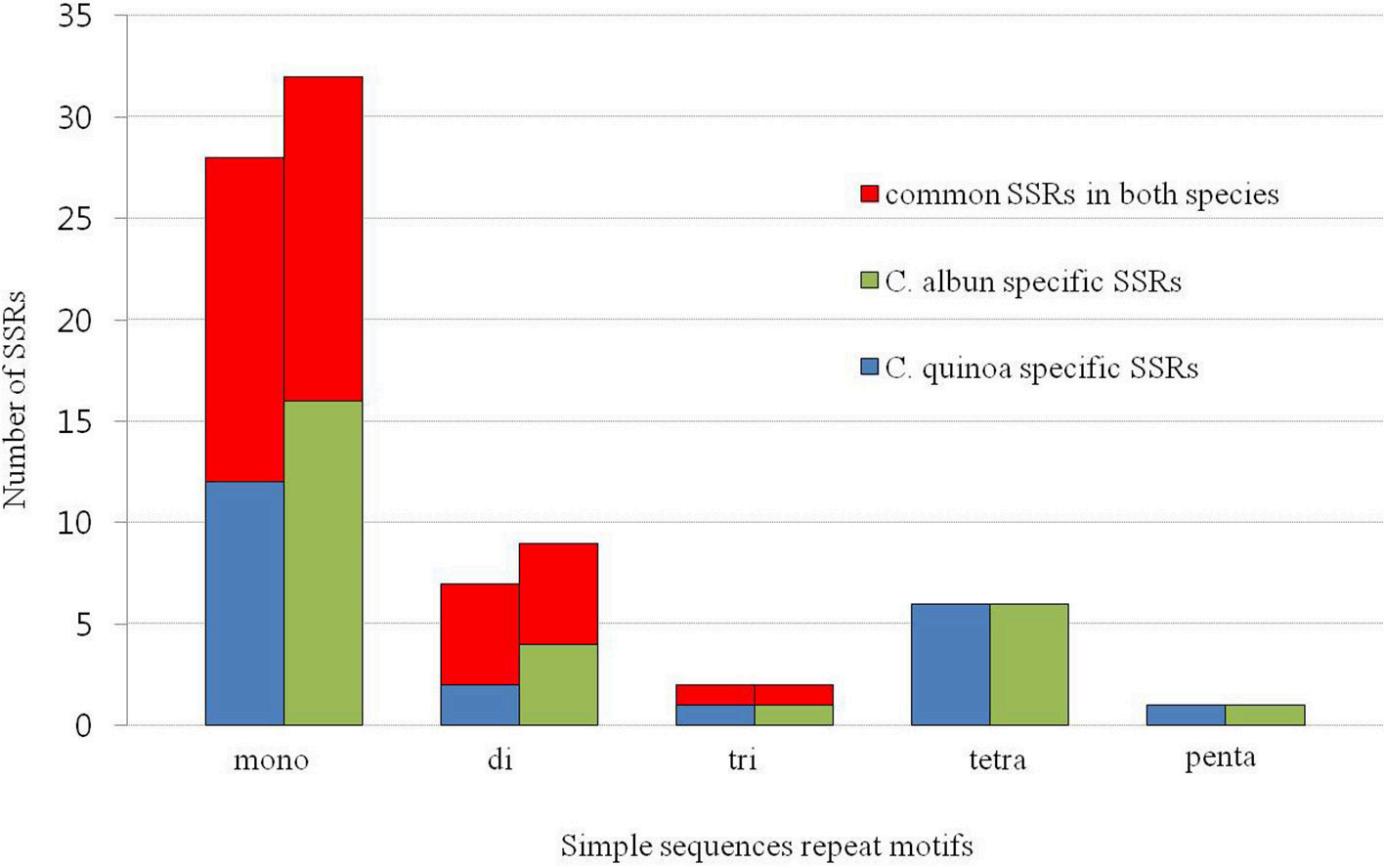
Frequency of simple sequence repeats (SSRs) in the chloroplast genome of two *Chenopodium* species.

We identified 44 and 53 SSRs in the cp genome of *C. quinoa* and *C. album*, respectively (Table S7). The most abundant SSRs motifs were mononucleotides, accounting for about 62 and 66% of the SSRs motifs in *C. quinoa* and *C. album*, respectively, and the majority repeat sequence was A/T. A total of 28 SSRs were shared by both species and they were mostly detected in the LSC region, inter-genic sequences, and mononucleotides (Figure 5).

### *trnI-GAU* intron sequence variation in chenopodioideae

**Figure 6:**
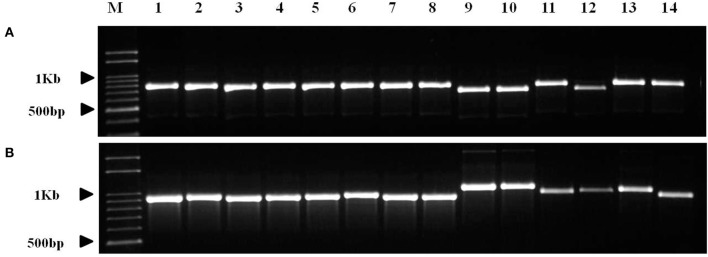
PCR amplification of *Chenopodium quinoa* germplasm and seven *Chenopodium* species using InDel markers. **(A)** InDel_QA_01; **(B)** InDel_QA_02. Details of the germplasm list are shown in Table S1. 1–8, *Chenopodium quinoa*; 9, *C. album*; 10, *C. koraiense*; 11, *C. glaucum*; 12, *C. ficifolium*; 13, *C. hybridum*; 14, *C. pumilio*.

**Figure 7:**
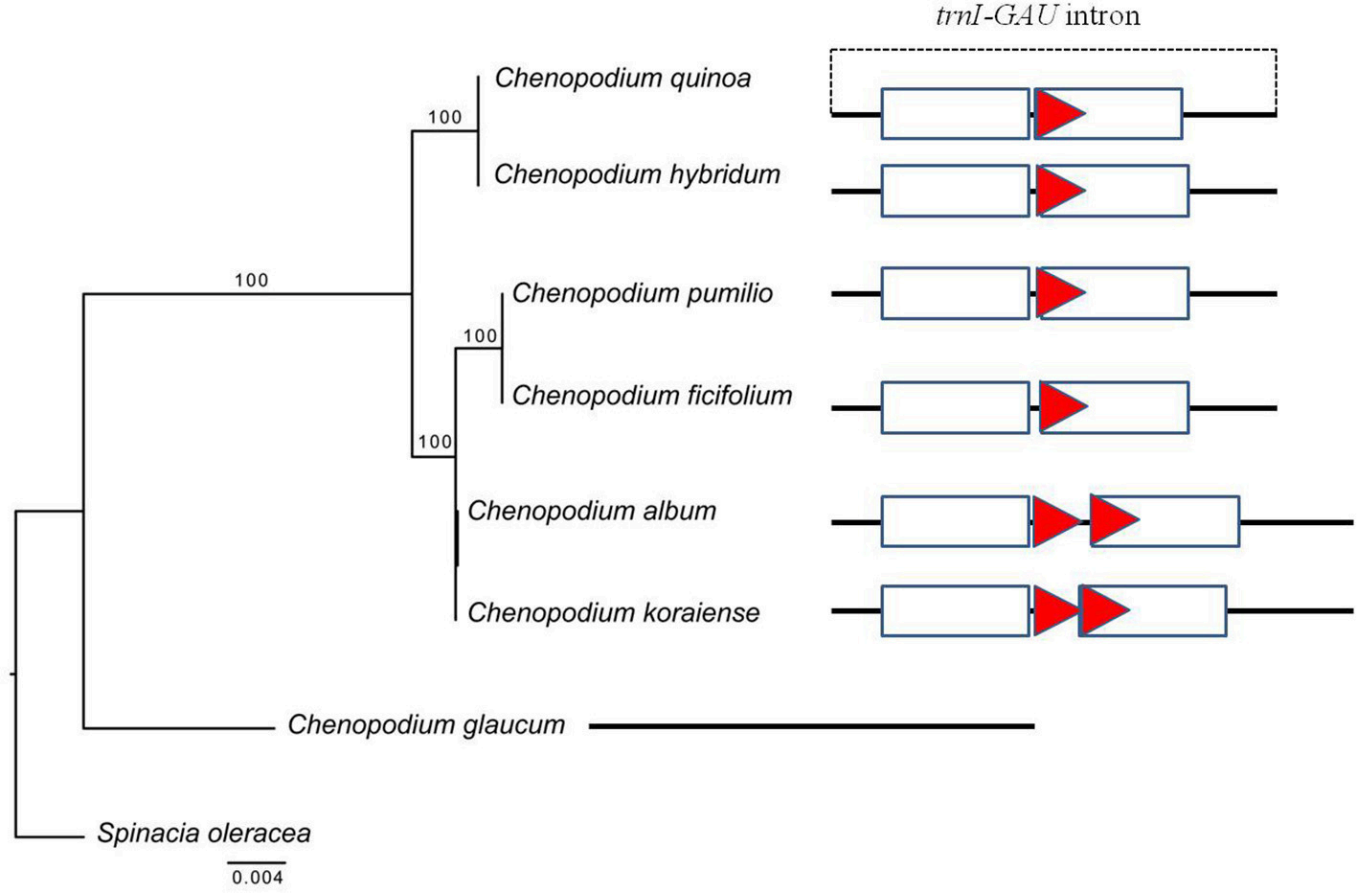
Phylogenetic tree reconstruction and copy number variation of tandem repeats in eight taxa using maximum likelihood analysis based on *trnI-GAU* sequences. Bootstrap values >50% are given at the nodes. The triangle indicates tandem repeat (66 bp) and sequence information for each taxon is shown in Figure S3. The rectangle represents tandem repeats (95 bp) in the *trnI-GAU* gene.

The copy number variation of TRs in *trnI-GAU* intron sequences among Chenopodioideae was also investigated (Figure 6). The total length of the *trnI-GAU* intron in eight species, seven *Chenopodium* species and one outgroup, ranged from 805 bp (*S. oleracea*) to 1,109 bp (*C. album* and *Chenopodium koraiense*); the length of aligned sequences was 996 bp (Table S8; Figure S4). *C. album* and *C. koraiense* possessed two copies of TR10 (66 bp), four species (*C. quinoa, Chenopodium hybridum, Chenopodium pumilio, Chenopodium ficifolium*) had one copy, and *Chenopodium glaucum* had no TR10 in the *trnI-GAU* sequences. All *Chenopodium* species, except for *C. glaucum*, contained two copies of TR11 (95 bp) in the *trnI-GAU* sequences (Table 3). The maximum likelihood analysis resolved *Chenopodium* monophyletic. *C. glaucum* was the earliest diverging lineage and sister to other species. *C. album* and *C. koraiense* formed a clade that was sister to the *C. pumilio* and *C. ficifolium* clade. *C. quinoa* clustered together with *C. hybridum* in a strongly supported clade (boostrap support = 100; Figure 7).

**Table 3:** Copy number variation of tandem repeats and intron size of *trnI-GAU* gene in chloroplast genome sequences of the seven Chenopodium taxa with out-group (*Spinacia olreacea*).

**Species**	**Copy number of tandem repeat (TR10^z^)**	**Intron size (bp)**
*Chenopodium quinoa*	1	1,043
*Chenopodium album*	2	1,109
*Chenopodium koraiense*	2	1,109
*Chenopodium glaucum*	0	1,030
*Chenopodium ficifolium*	1	1,043
*Chenopodium hybridum*	1	1,043
*Chenopodium pumilio*	1	1,043
*Spinacia oleracea*	0	805

z *TR10 information is shown in Table 2*.

### Phylogenetic relationship of 59 protein-coding genes in the cp genome

**Figure 8:**
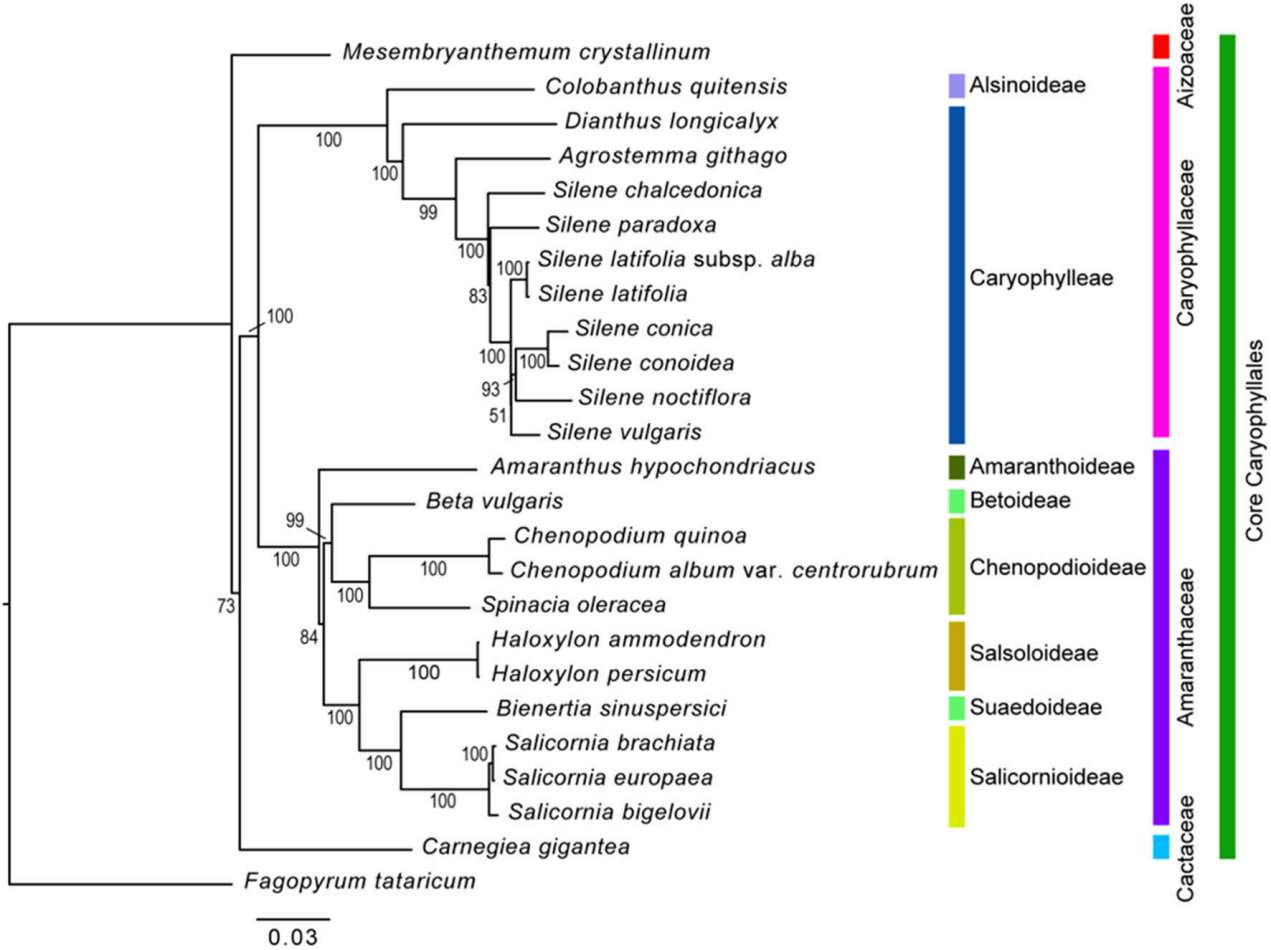
Phylogenetic tree reconstruction of 25 taxa using maximum likelihood based on 59 protein-coding genes. Bootstrap values >50% are given at the nodes.

The maximum likelihood analysis was conducted based on 59 protein-coding genes from 25 taxa (Figure 8). The length of aligned protein-coding gene sequences was 48,361 bp. In the phylogenetic tree, the Core Caryophyllales were monophyletic and formed four clades. Aizoaceae (*Mesembryanthemum crystallinum*) occupied the most basal position, followed by Cactaceae (*Carnegiea gigantea*). In the Caryophyllaceae clade, Alsinoideae (*Colobanthus quitensis*) were a sister to Caryophylleae. Amaranthaceae formed three subclades: Amaranthoideae (*Amaranthus hypochondriacus*) were the most basal and sister to the remaining five subfamilies; Salicornioideae, Suaedoideae, and Salsoloideae formed a clade; and Betoideae (*Beta vulgaris*) was sister to Chenopodioideae. Within Chenopodioideae, the sister relationship between *S. oleracea* and *Chenopodium* (*C. quinoa* and *C. album*) was highly supported (bootstrap support = 100).

## Discussion

### Comparative analysis of the *Chenopodium* chloroplast genome

The complete cp genome sequences provide valuable information in plant phylogenies due to their highly conserved genome structure and higher evolutionary rate as compared to that of the mitochondrial genome (Chaney et al., 2016). Although, the cp genome has a nearly collinear gene order in most land plants, the changes in the genome such as sequence inversion (Cho et al., 2015), gene loss (Fu et al., 2016), and expansion at the borders of the LSC, SSC, and IR regions (Choi et al., 2016) occur in the course of evolution. We found a 3.1 kb inversion in the *rbcL* to *trnV* region of the *Chenopodium* cp genome when its sequences were compared to the sequences of *S. oleracea*; this inversion may have been facilitated by tRNA activity (Walker et al., 2014) or by high G + C content (Fullerton et al., 2001). The flanking region of the inversion contained a tRNA gene, including intron sequences with similar G + C content (37.98%), indicating that the 3.1 kb inversion may be promoted by the presence of the tRNA. The border regions between two IR regions and the SSC region have contributed to genome size variation by expansion or contraction among land plants (Cho and Park, 2016; Hu et al., 2016; Ni et al., 2016). Although, the genome size differs between *C. album* and *C. quinoa*, the results of the present study revealed that the junction areas were highly conserved.

Repeat sequences such as TRs and SSRs play an important role in the rearrangement and stabilization of cp genome sequences (Vieira et al., 2014) and the copy number variation in different species, even in the same species (Kim et al., 2015), which characteristics render them suitable molecular markers for authentication (Cho et al., 2015, 2016) and phylogenetic analysis (Yang et al., 2013; Williams et al., 2016). The occurrence of the repeats is more prevalent in the intergenic sequence than it is in the CDS, which was also confirmed in this study (Table 2; Table S7). TRs and SSRs are possibly related to cp genome size variation and divergence because of the recombination (Ogihara et al., 1988; Marshall et al., 2001). In this study, the SSRs and TRs were prevalent in the LSC region and contributed to 68 bp longer genome of *C. album* compared to that of *C. quinoa*.

### Divergence region of the *Chenopodium* chloroplast genome

In previous molecular phylogenetic studies, *Chenopodium* formed a polyphyletic group and phylogenetic relationships of some of the taxa were unclear (Kadereit et al., 2003, 2010; Fuentes-Bazan et al., 2012b). These studies were based on the ITS sequences of the nuclear ribosomal DNA and *trnL-trnF, matK-trnK, atpB, atpB-rbcL*, and *rbcL* sequences of the cp genome. In the present study, the nucleotide diversity of the cp regions was relatively low (*trnL-trnF*, 0.01918; *matK*, 0.00982; *trnK-UUU* intron, 0.01359; *atpB*, 0.00601; *atpB-rbcL*, 0.00689; *rbcL*, 0.00493). Based on our study, high sequence divergence was detected in the following regions: *psbK-psbI, psbI-trnS, ycf3-trnS, trnS-rps4, rps4-trnT, trnT-trnL, trnM-trnV, cemA-petA, psbJ-psbL, trnW-trnP, psaJ-rpl33, petD-rpoA, rpl16-rps3, rpl22-rps19, rrn23-rrn4.5, ccsA-ndhD*, and *rpl32-trnL* (Figure 3; Table S4). Therefore, these regions are considered useful markers for elucidating the phylogenetic relationship within *Chenopodium*. However, when selecting suitable molecular markers, the length of amplified regions must also be considered. The length of nine regions, *psbI-trnS, trnM-trnV, psbJ-psbL, trnW-trnP, petD-rpoA, rpl16-rps3, rpl22-rps19, rrn23-rrn4.5*, and *ccsA-ndhD*, is considered relatively short and insufficient to reproduce the nucleotide variation in various taxa. In contrast, the remaining eight regions (*psbK-psbI, ycf3-trnS, trnS-rps4, rps4-trnT, trnT-trnL, cemA-petA, psaJ-rpl33*, and *rpl32-trnL*) are judged suitable for phylogenetic analysis of *Chenopodium* and helpful to evaluate unresolved phylogenetic relationships.

### Intron sequence variation in *Chenopodium* species

Introns in cp genomes are generally conserved, but structural variations such as sequence loss or variations (SNP), have been reported in several species. Structural intron variation is known to occur in ATP synthetase (*atpF*), RNA polymerase (*rpoC2*), and ribosomal proteins (*rpl2, rps12*, and *rps16*; Daniell et al., 2016; He et al., 2017). Introns have important roles in gene expression regulation by alternative splicing or stabilization of transcripts and they are gained or lost over evolutionary time (Daniell et al., 2008). Intron variations are also often implemented in phylogenetic and evolutionary analyses. In the present study, we identified 10 proteins and 6 tRNAs with introns in cp genes (Table S3). Although intron sequence variation such as transversion, transition, and small InDels (3–10 bp) have been reported in proteins (Cho et al., 2016; Devi and Chrungoo, 2017), the present study is the first report of the variations in TR copy number in tRNA introns. The changes in highly conserved cp genes have been used to resolve phylogenetic relationships in angiosperm families. To test whether our findings can be applied in phylogenetic analysis, we investigate the copy number variation of the *trnI-GAU* intron in other *Chenopodium* species in Korea. All the seven *Chenopodium* species, except *C. glaucum*, contained the same TR motifs and copy number variations. These results implied that *trnI-GAU* intron sequences provide valuable information about *Chenopodium* phylogenetic relationships. Additional studies should examine whether the copy number variation is present in other *Chenopodium* species and explore other properties such as transcript stability of the cp genome among different *Chenopodium* species.

### Comparison of phylogenetic relationships with previous studies

The results of the phylogenetic analysis using 59 protein-coding genes of 24 Core Caryophyllales species and one outgroup resulted in a well-resolved topology in which the monophyly of the tested families and subfamilies was supported. However, our results showed a slight difference from the APG IV system (The Angiosperm Phylogeny Group, 2016). Specifically, Aizoaceae were placed in the most basal clade and Cactaceae formed a sister clade to Caryophyllaceae and Amaranthaceae. In contrast, Caryophyllaceae and Amaranthaceae are in a clade sister to other two families in the APG IV system. In addition, the phylogenetic relationships among Amaranthaceae species in the present study did not corroborate the results of the previous study based on *rbcL* sequences (Kadereit et al., 2010): (1) Amaranthoideae formed a basal clade within the Amaranthaceae; (2) Betoideae were sister to Chenopodioideae, but they formed an unresolved paraphyletic clade in the previous study; and (3) Chenopodioideae were more closely related to Betoideae, instead to Salsoloideae, Suaedoideae, and Salicornioideae reported in the previous study. We believe that these differences are due to increased resolution resulting from the addition of more gene regions. However, the present study analyzed a limited number of species. Therefore, further studies should include various species to further elucidate the phylogenetic relationships of Caryophyllales and Amaranthaceae.

## Author contributions

SH and JS conceived the design of the study, analyzed the data and drafted the manuscript. KC and HL performed the bioinformatics work. KY collected and identified samples. SK, JN, HS, YK grew and collected samples of *Chenopodium quinoa* germplasm in HARI. KC was responsible for data analysis and writing of the manuscript. All authors read and approved the final manuscript.

### Conflict of interest statement

The authors declare that the research was conducted in the absence of any commercial or financial relationships that could be construed as a potential conflict of interest.

## Supplementary material

Figure S1 BLASTZ analysis of *Chenopodium quinoa* chloroplast genome against *Spinacia oleracea* (NC_002202) chloroplast sequences. The inversion region is delimited with the red rectangular line. Blue and yellow bars indicate contigs matching the reference sequence in forward and reverse orientation, respectively.

Figure S2 Comparison of the chloroplast genomes between *Chenopodium quinoa* and *C. album* using mVISTA LAGAN program. Blue block: conserved gene; sky blue: tRNA and rRNA; red block: intergenic region. White regions indicate sequence divergence between two chloroplast sequences.

Figure S3 Dot-plot analysis and sequence comparison of InDel_QA_02 region between *Chenopodium quinoa* and *C. album.* The Indel_QA_02 region is shown in Figure 4. Tandem repeats are underlined. *C. album* has two tandem repeat units, whereas *C. quinoa* has one unit.

Figure S4 ClustalW alignment of trnI-GAU gene intron sequences of the chloroplast genome from seven Chenopodium species.

## Abbreviations

CDS: coding sequences
cp: chloroplast
IRs: inverted repeats
LSC: large single copy region
SSRs: simple sequence repeats.
