# Supplementary figures and images for: Complete Chloroplast Genome Sequences and Comparative Analysis of Chenopodium quinoa and C. album
Source: Front Plant Sci. 2017 Oct 6;8:1696. doi: 10.3389/fpls.2017.01696 (PMC5635682; doi:10.3389/fpls.2017.01696)

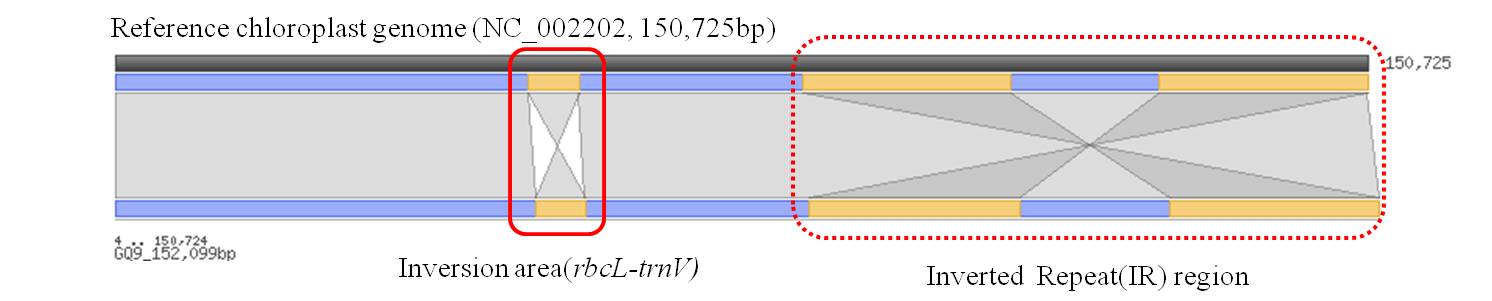

Supplement: Figure S1 — BLASTZ analysis of Chenopodium quinoa chloroplast genome against Spinacia oleracea (NC_002202) chloroplast sequences. The inversion region is delimited with the red rectangular line. Blue and yellow bars indicate contigs matching the reference sequence in forward and reverse orientation, respectively. [file Image1.jpg]

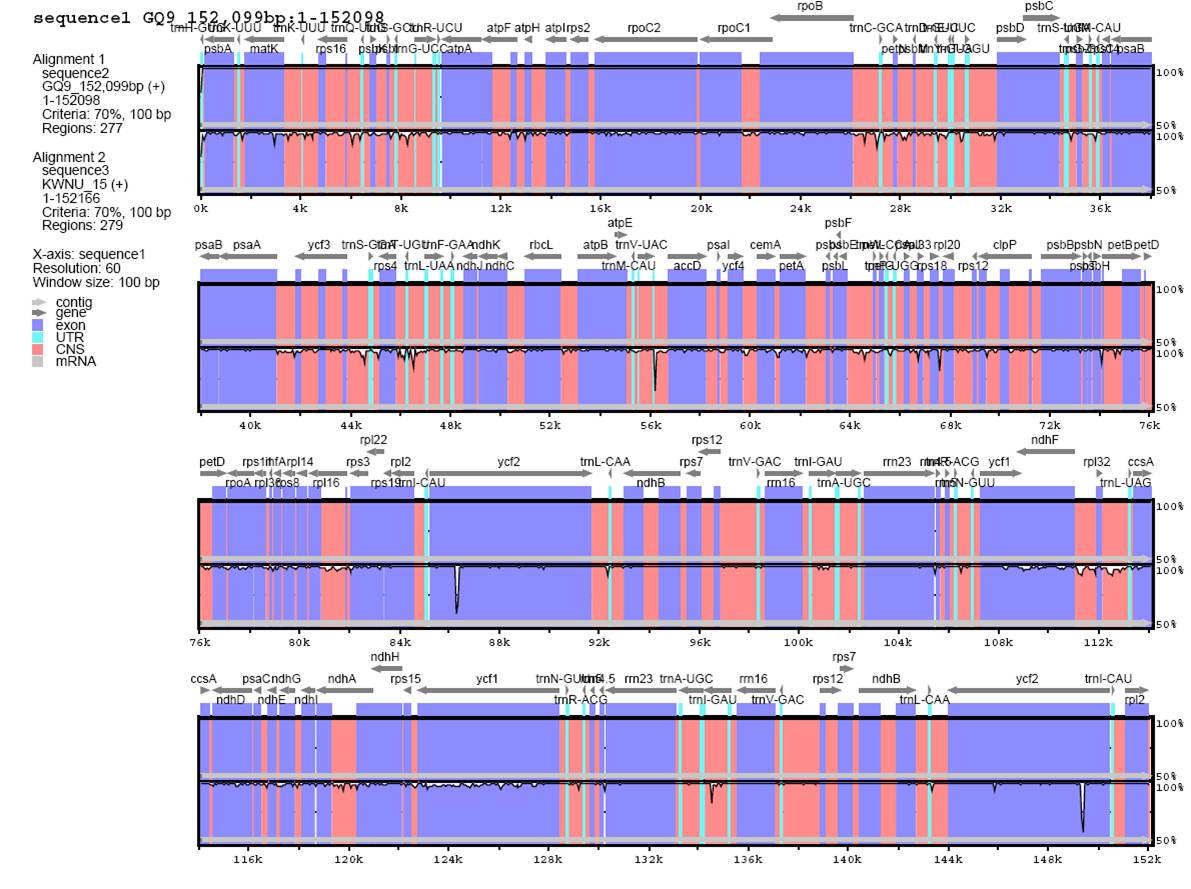

Supplement: Figure S2 — Comparison of the chloroplast genomes between Chenopodium quinoa and C. album using mVISTA LAGAN program. Blue block: conserved gene; sky blue: tRNA and rRNA; red block: intergenic region. White regions indicate sequence divergence between two chloroplast sequences. [file Image2.jpg]

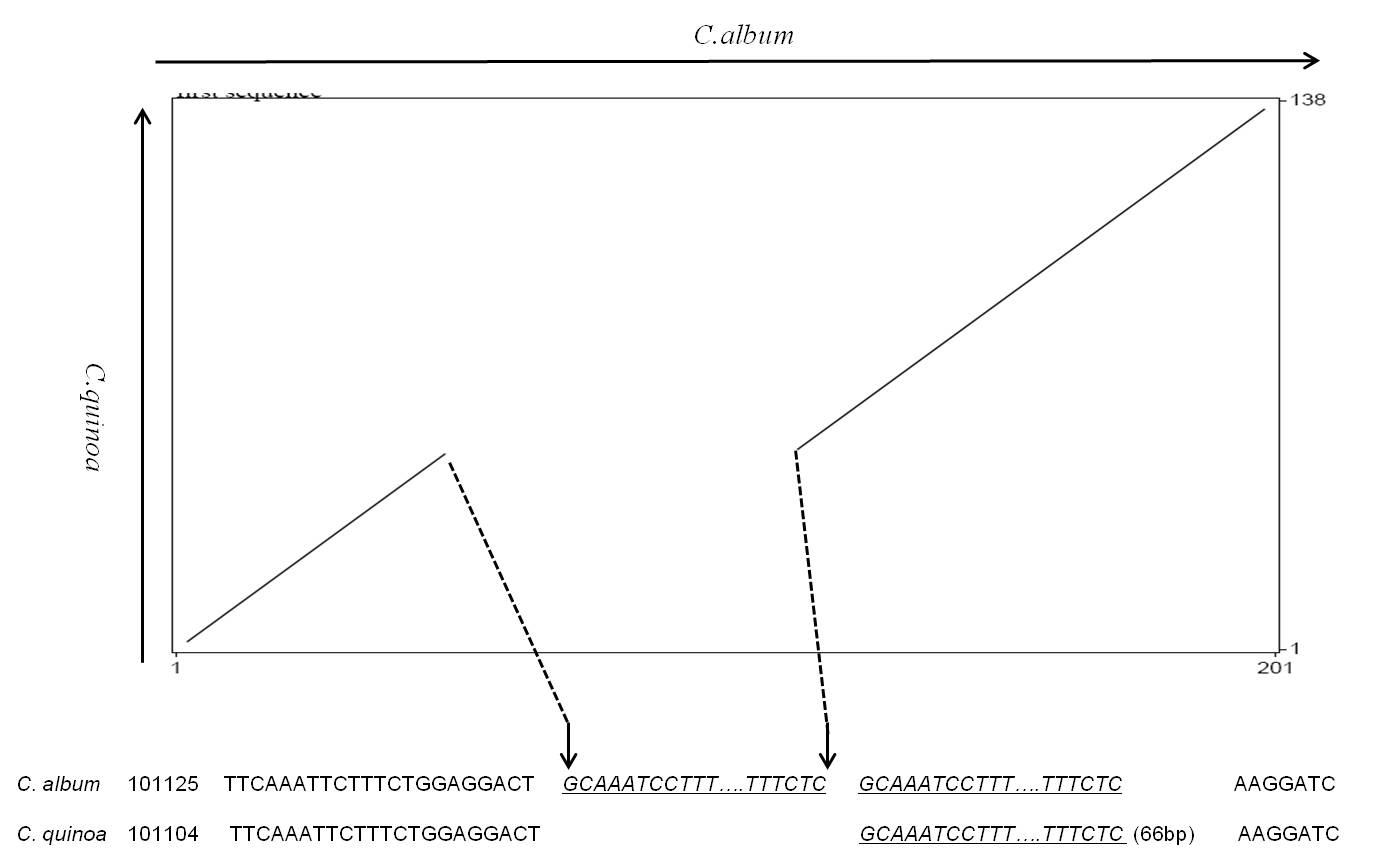

Supplement: Figure S3 — Dot-plot analysis and sequence comparison of InDel_QA_02 region between Chenopodium quinoa and C. album. The Indel_QA_02 region is shown in Figure 4. Tandem repeats are underlined. C. album has two tandem repeat units, whereas C. quinoa has one unit. [file Image3.jpg]

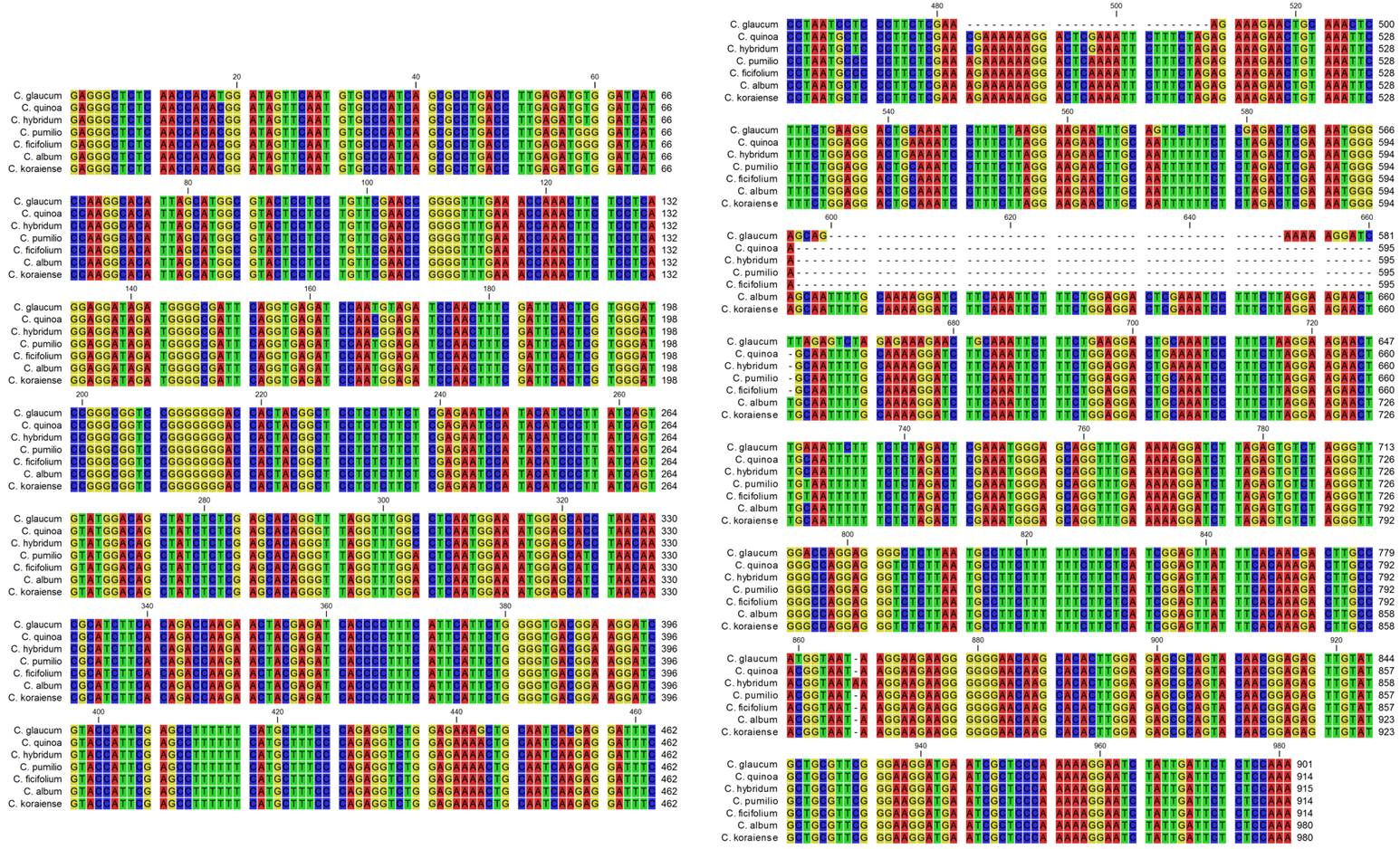

Supplement: Figure S4 — ClustalW alignment of trnI-GAU gene intron sequences of the chloroplast genome from seven Chenopodium species. [file Image4.jpg]
